# Resource Allocation in the Pediatric Intensive Care Unit in Rwanda

**DOI:** 10.5334/aogh.4714

**Published:** 2025-08-26

**Authors:** Tracy Kelly, Owen Selden, Dazhanae Houston, Derek Meyers, Brenna Kent, Aimable Kanyamuhunga

**Affiliations:** 1 University of Virginia, Charlottesville, Virginia, USA; 2 University of Rwanda College of Medicine and Health Sciences, Pediatrics & Child Health Department, Kigali, Rwanda

**Keywords:** pediatrics, critical care, resource allocation, low resourced, health system

## Abstract

*Background:* Children born in low‑ and middle‑income countries are 14 times more likely to die before reaching the age of five compared to children in high‑income countries. Pediatric Intensive Care Units (PICUs) with specialized equipment and advanced medications managed by trained clinicians have reduced mortality of children worldwide, yet countries with limited funds and scarce resources strain to meet needs of critically ill children.

*Objectives:* The aim of the study was to identify the disease burden of patients entering the PICU at the Central Hospital in Kigali, Rwanda, and the relationship between patient mortality and allocation of resources. In addition, this study focused on several factors suspected to impact the mortality rate, including the entry point into the health system, delay in admittance, and whether surgery was performed.

*Method:* A retrospective, cross‑sectional review of 30 medical records per year was conducted between January 2016 and December 2022, totaling 177 encounters. Demographic and clinical data were extracted and analyzed to perform descriptive and inferential statistics, including univariable and multivariable logistic regression analyses to identify factors affecting mortality.

*Findings:* The study showed an overall mortality rate of 55% for patients admitted to the PICU. Among patients who died, the most common diagnoses were sepsis, primary respiratory failure, and congenital defects. When holding age and surgery constant, patients with a noted delay in admittance to the PICU had increased odds of mortality than those without a delay. Holding the delay in admittance constant, there was an interaction effect between age and surgery on mortality, with higher odds of mortality in newborns than in children over one month of age when surgery was performed.

*Conclusions:* Careful adherence to emerging pediatric sepsis guidelines, immediate recognition, and appropriate treatment may reduce mortality. Prioritizing policies that reduce delays in treating critically ill children may improve outcomes.

## Background

Over the last several decades, global childhood mortality has declined significantly due to emphasis on the importance of primary care, early diagnostic testing, and attention to protecting children from accidents. In addition, the rapid expansion of intensive care units designed uniquely for children has helped to increase the survival of critically ill children [1]. With trained physicians and nurses, sophisticated equipment and ventilatory support, and powerful medications to combat infection and shock, children who otherwise may have died are now surviving. Coordinated transfer of patients from one health facility to another with inter‑hospital communication networks, computerized records, and digital transfer of critical data ensures rapid care from a referral facility to the receiving hospital. However, there is an inherent inequity in the availability and access to Pediatric Intensive Care Units (PICU) between wealthy countries and less resourced countries. This study exemplifies the importance of the global health priority of equalizing care for critically ill children worldwide. According to the 2022 report by the United Nations Inter‑agency Group for Child Mortality, children born in low‑ and middle‑income countries (LMICs) are 14 times more likely to die before reaching the age of five compared to children in high‑income countries [2]. Furthermore, the global burden of disease is heavier in countries where infectious diseases, including malaria, pneumonia, and diarrheal diseases, are the leading cause of childhood death [3]. Increased mortality was seen globally during the COVID‑19 pandemic when excessive burden to hospital capacity was caused by an imbalance in the supply–demand ratio of clinical and human resources [4]. This situation of staff shortages, infrastructure limitations, and inadequate supply chains exists continuously in less resourced countries, contributing to inequities in outcomes. The aim of this study was to identify the disease burden of patients entering the PICU and the relationship between patient mortality and allocation of resources. In addition, this study focused on several factors that were suspected to impact the mortality rate.

## Literature Review

PICUs are designed to provide lifesaving, resuscitative, and critical interventions to children. Yet the cost associated with training physicians and nurses, building and supplying facilities, and maintaining supplies has limited the development of these expensive units in low‑income countries [5]. When properly staffed and managed with needed essential resources, low‑cost interventions can effectively treat critically ill patients [6]. Grouping patients who require the most serious medical intervention in one setting allows for greater capacity to concentrate resources for treatment through the intervention of specially trained nurses and pediatric intensivists [7]. In a cross‑sectional study at the PICU of St. Paul’s Hospital, Addis Ababa, Ethiopia, the positive management of a PICU by a pediatric intensivist yielded a twofold increase in patients admitted and treated [7].

Health provider density, specialized education, and the ratio of critical care beds to population are significant factors for patient outcomes. Many hospitals in low‑resourced countries either lack a PICU altogether or have limited capacity for the population [5]. In a 2015 study of intensive care unit capacity, most LMICs had no critical care services or lacked any published data, and those that did had insufficient bed capacity for their population [8]. No country in the study had more than 10 beds devoted exclusively to the care of critically ill children. The country of Rwanda, with a population of 13.5 million (40% of whom are under 16 years old), has 0.2 PICU beds per 100,000 children. For context, there are 6.8 to 8.8 PICU beds per 100,000 children in the United States [9]. In addition, many hospitals lack adequate healthcare workers to properly care for a large census of critically ill children [8]. While the number of physicians worldwide has increased, there remains a significant disparity between the number of physicians in Sub‑Saharan African countries and higher income countries [10]. In PICUs where adequate numbers of staff are present, the attending physician often has not had specialized training in pediatric intensive care, negatively affecting mortality rates [8].

A study conducted in a Southeast African country found the mortality rate in a mixed adult and PICU to be 53% [11]. The authors concluded that training of pediatric intensive care specialists was needed to properly care for critically ill children in Malawi. Another paper found the overall mortality rate of a newly opened PICU in Rwanda to be 50% with male gender, use of vasoactive medications, discharge diagnosis of septic shock, and malnutrition upon admission as the most significant contributors to mortality [10]. Given the high mortality outcomes in PICUs in LMIC, researchers have studied tools and methods to optimize care and monitor patients with deteriorating conditions [12]. The Pediatric Early Warning Score for Resource‑Limited Settings (PEWS‑RL) has been identified as one resource to help identify children at risk for clinical deterioration and promote better interprofessional collaboration between nurses and physicians [13, 14].

Treatment in the PICU is urgent and, in most instances, such as trauma, should be initiated immediately. There are multiple reasons for delays that endanger survival. A recent study on barriers to injury care in Rwanda noted four levels of delays that involve seeking care, reaching care, receiving care, and remaining in care [15]. The pediatric population is especially vulnerable to delays in critical care due to their small size and rapid decline with sepsis.

## Methodology

### Study design

A retrospective chart review and analysis were conducted at the PICU at the University Teaching Hospital of Kigali (CHUK). Ethical and scientific approval was sought and received from the Ethics Committee Institutional Review Board at both the University of Virginia IRB (study number 24585) and the University of Rwanda Ethics Committee (study number EC/CHUK/102/2023), and both waived written informed consent. Access to the medical records was approved by the Research office at CHUK. Patient confidentiality was maintained using a random study ID, and no identifying health information was transferred outside the hospital.

### Study setting

Rwandan healthcare is ordered in a three‑tiered pyramid structure. Primary care is provided by the community health centers and basic health posts, secondary care is at the district and provincial hospitals, and tertiary care is provided by the country’s five referral teaching hospitals, one of which is CHUK. The Community Based Health Insurance (CBHI), also known as Mutuelle de santé, is the most common health plan as it aims to provide universal health coverage through allowing members to pay a contribution dependent on the number of people in their households to receive health coverage at hospitals across the country [16]. Over 91% of the population participates, and nearly all patients fall within the middle range of contribution. There are no extreme variables in income brackets within the country, yet according to the World Bank, half the population remains below the international poverty line [17]. The Pediatric Department of the CHUK provides both in and outpatient care across specialty services, including surgery, oncology, high dependency respiratory care, cardiac care, nephrology, and malnutrition. The department has an inpatient capacity of 86 beds, including a standalone three‑bed PICU, with a fourth emergency bed that opened in August 2012 [18]. Criteria for admission to the PICU include pulmonary and airway support with invasive ventilation, infusion of inotropes, post‑operative recovery, and other clinical conditions requiring ventilatory support. The unit has basic critical care equipment without ECMO, ventricular assist device (VAD), dialysis, or other intensive interventions. The PICU prioritizes, receives, and provides care to patients from within the hospital and those referred to from outside the hospital.

### Data collected

A simple random sample using Excel of 30 patient records per year of children who entered the PICU between January 2016 and December 2022 was reviewed. Most patient records were handwritten paper charts and manually reviewed by the researchers. Inclusion criteria included all patients admitted to the PICU and whose records were available for review. Exclusion criteria included missing records and records of children who arrived at the PICU postmortem. After accounting for duplications and exclusion criteria, 177 visit observations were reviewed.

Data were collected on sociodemographic characteristics, clinical variables, and mortality. The following sociodemographic information was captured: gender, age, weight at the time of admission, region of primary residence, and health insurance type. Age was standardized into months, as most patients were under two years old. To focus on the most sensitive age group, age was also converted to a binary variable to indicate whether patients were younger or older than one month.

Clinical variables included the time between the onset of symptoms and hospitalization, entry unit prior to admission to PICU, transfer time between initial hospital and PICU, diagnosis (at arrival, admission, and discharge), length of stay in PICU, whether surgery was performed, and mortality.

In addition, researchers documented whether a delay in PICU admission was explicitly noted in the patient’s chart. This variable does not provide insight into the length of delay or delays that occurred before entering the health system, such as seeking alternative traditional treatment.

All information was recorded at the individual visit level, meaning there could be multiple PICU admissions per patient. Not all variables were always available or complete.

### Categorization of diagnoses and medications

Diagnoses and prescribed medications were provided in text fields. Both fields often used alternative spellings of diagnoses and medications. To account for this, string matching, using the stringr package from R software [19, 20], was used to categorize and summarize the diagnoses and medications. Verifying the validity of the clinician’s diagnosis in accordance with current pediatric guidelines was beyond the scope of this paper.

Because of the diversity of potential diagnoses, they were grouped into categories before analysis. These categories included primary respiratory failure, congenital defects, renal failure, sepsis, trauma, neurological insult, cancer, malnutrition, and systemic infections. It is possible for a diagnosis to match multiple categories, such as a patient with sepsis and malnutrition. The full list of words and phrases used to assign categories can be found in the supplemental table.

A similar method was applied to prescribed medications. Medications were grouped into broad categories of anti‑infective agents, sedatives, resuscitation/inotropes, antipyretics, antiepileptics, diuretics, and nutritional electrolyte supplements. Antibiotic usage was of interest, so they were examined individually.

### Statistical analysis

Data were analyzed using R software using the package ggplot2 for graphs and the packages gt and table 1 for tables [21, 22]. Descriptive analyses of attributes collected for visits were performed. The median and interquartile range (IQR) were reported for quantitative variables. Categorical variables were summarized as counts and percentages. For both types of variables, the number of missing values was also recorded.

**Table 1:** Sociodemographic characteristics.

	≤ 1 MONTH (*N* = 61)	> 1 MONTH (*N* = 116)	OVERALL (*N* = 177)
**Weight (kg)**			
Median [IQR]	2.5 [1.7, 3.0]	11.3 [7.0, 21.8]	5.1 [2.8, 15.0]
Missing (%)	0 (0)	10 (8.6)	10 (5.6)
**Gender, *n* (%)**			
1: Male	33 (54.1)	68 (58.6)	101 (57.1)
2: Female	28 (45.9)	45 (38.8)	73 (41.2)
Missing	0 (0)	3 (2.6)	3 (1.7)
**Region of Residence, *n* (%)**			
1: Kigali	20 (32.8)	36 (31.0)	56 (31.6)
2: Southern Province	12 (19.7)	18 (15.5)	30 (16.9)
3: West Province	4 (6.6)	16 (13.8)	20 (11.3)
4: North Province	15 (24.6)	29 (25.0)	44 (24.9)
5: East Province	10 (16.4)	12 (10.3)	22 (12.4)
6: Other	0 (0)	1 (0.9)	1 (0.6)
Missing	0 (0)	4 (3.4)	4 (2.3)
**Health Insurance, *n* (%)**			
CBHI Insurance	56 (91.8)	106 (91.4)	162 (91.5)
Private Health Insurance	1 (1.6)	0 (0)	1 (0.6)
Rwandaise d’Assurance Maladie (RAMA) and MMI	0 (0)	1 (0.9)	1 (0.6)
Missing	4 (6.6)	9 (7.8)	13 (7.3)

Univariable and multivariable logistic regression techniques were used to examine relationships between information available at admission to the PICU and mortality. To ensure the assumption of independence, eight visits from four patients with repeated admissions were removed.

The following variables available at admission were first tested with univariable logistic regression: gender, age, weight, region of primary residence, time between symptom onset and hospitalization, transfer time between initial hospital and the PICU, surgery, and delay in admission. The relationship between diagnoses at admission and mortality was also examined with univariable logistic regression. However, admission diagnoses were not considered for the multivariable model due to limitations in how they were recorded. There was substantial variation in how diagnoses were recorded, and patients often had co‑occurring diagnoses, complicating their interpretation. Complete case analysis was used for the univariable logistic regressions, so they had variable sample sizes.

Variables that were significant at *p* < 0.05 in the univariable analyses were considered for inclusion in the multivariable logistic regression model. An interaction between age and surgery was also tested since surgery performed within the newborn period is often more critical for survival. Multicollinearity and outlier concerns were checked with variance inflation factors and Cook’s distance, respectively. Four observations had Cook’s distances greater than 4/*n*, all involving patients with delayed admission who survived. Since they represented a unique outcome, they were maintained within the model. All independent variables in the final regression model were categorical, so there was no need to confirm that there was a linear relationship between independent variables and the logit of the dependent variable.

Post hoc analysis was performed for the multivariable logistic regression, using the effects [23–25] and emmeans packages in R [26]^.^ The Tukey method for comparison between multiple groups was applied to perform pairwise comparisons on the estimated marginal means (EMMs). The level of statistical significance for all comparisons was 0.05, and 95% confidence intervals were calculated.

## Findings

### Demographic overview

Within the dataset of the 177 PICU patient visits, patients ranged in age from newborns to 15 years old. However, the majority of patients were under one year of age, with a median age of 9 months. Males accounted for 57% of the patient visits. Representation by region varied with the Kigali region, where the hospital is located, as the most represented with 32% of observations, followed by 25% from the Northern Province and 17% from the Southern Province. The Community Based Health Insurance (CBHI) was identified in all but two patients (Table 1).

Based on the data available, the median time between symptom onset and hospitalization was three days (IQR 0–7). The median transfer time between initial hospital and admission into the PICU was three days (IQR 1–9.8). After entering CHUK, patients were most likely to be triaged through the emergency room, accounting for 45% of observations. Transfers from the general unit accounted for 20% of observations, and the remaining cases came from a variety of locations, such as neonatology or the operating theater. Once admitted, the median length of stay in the PICU was five days (IQR 2‑14). Ultimately, 55% of cases ended in the death of a patient (Table 2).

**Table 2:** Clinical characteristics.

	≤ 1 MONTH (*N* = 61)	> 1 MONTH (*N* = 116)	OVERALL (*N* = 177)
**Time between symptom onset and hospitalization (days)**			
Median [IQR]	0.0 [0.0, 4.0]	4.5 [2.0, 21.0]	3.0 [0.0, 7.0]
Missing (%)	2 (3.3)	22 (19)	24 (13.6)
**Time between initial hospital and PICU (days)**			
Median [IQR]	5.5 [2.0, 13.2]	2.0 [0.0, 7.0]	3.0 [1.0, 9.8]
Missing (%)	9 (14.8)	54 (46.6)	63(35.6)
**Length of stay (days)**			
Median [IQR]	5.0 [2.0, 13.0]	5.0 [1.0, 14.5]	5.0 [2.0, 14.0]
Missing (%)	0 (0)	4 (3.4)	4 (2.3)
**Entry unit, *n* (%)**			
1: Emergency Room	17 (27.9)	62 (53.4)	79 (44.6)
2: General Unit	17 (27.9)	19 (16.4)	36 (20.3)
3: Other	27 (44.3)	29 (25.0)	56 (31.6)
Missing	0 (0)	6 (5.2)	6 (3.4%)
**Surgery, *n* (%)**			
Yes	46 (75.4)	49 (42.2)	95 (53.7)
Missing	0 (0)	0 (0)	0 (0)
**Admittance Delay, *n* (%)**			
Yes	8 (13.1)	16 (13.8)	24 (13.6)
Missing	0 (0)	0 (0)	0 (0)
**Mortality, *n* (%)**			
Yes	38 (62.3)	59 (50.9)	97 (54.8)
Missing	0 (0)	0 (0)	0 (0)

It was of interest to understand how delays in admittance to the PICU affect patient mortality. Based on the information provided, 14% of patient visits had a delay in admittance to the PICU. The two most common documented reasons for a delay in admission to the PICU were occupancy of all available beds or lack of funds for transportation or laboratory exams. Additionally, the relationship between surgery and mortality was of interest, as many congenital defects will require surgery to decrease morbidity and improve survival. Surgery was common, occurring in 54% of patient visits (Table 2).

### Disease burden

To understand the disease burdens of the PICU, diagnosis information was examined. Diagnoses were recorded at both admission and discharge or death. On admission to the PICU, congenital defects and primary respiratory failure were the two most common diagnoses, accounting for 54 and 48 patient visits, respectively. At discharge or death, sepsis was the most listed diagnosis, accounting for 72 patient visits (Table 3).

**Table 3:** Diagnoses at admission and discharge/death.

DIAGNOSIS	ADMISSION	DISCHARGE
Congenital Defects	54	48
Primary Respiratory Failure	48	38
Systemic Infection	37	25
Sepsis	35	72
Neurological Insults	35	40
Cancer	15	12
Trauma	15	10
Malnutrition	11	15
Renal Failure	8	9
Other	12*^1^*	22*^2^*

*^1^*Includes one NA value.

*^2^*Includes three NA values.

At discharge, 46% of patients encountered more than one diagnosis, accounting for 34 unique combinations. Of the 34 combinations, 22 involved sepsis. The most common combinations of diagnoses were sepsis/primary respiratory failure and sepsis/congenital defects. Of patient encounters involving sepsis, 75% had at least one additional diagnosis and, on average, were included in 2.2 diagnosis categories, whereas patient visits not involving sepsis were included in 1.1 diagnosis categories on average.

**Figure 1:**
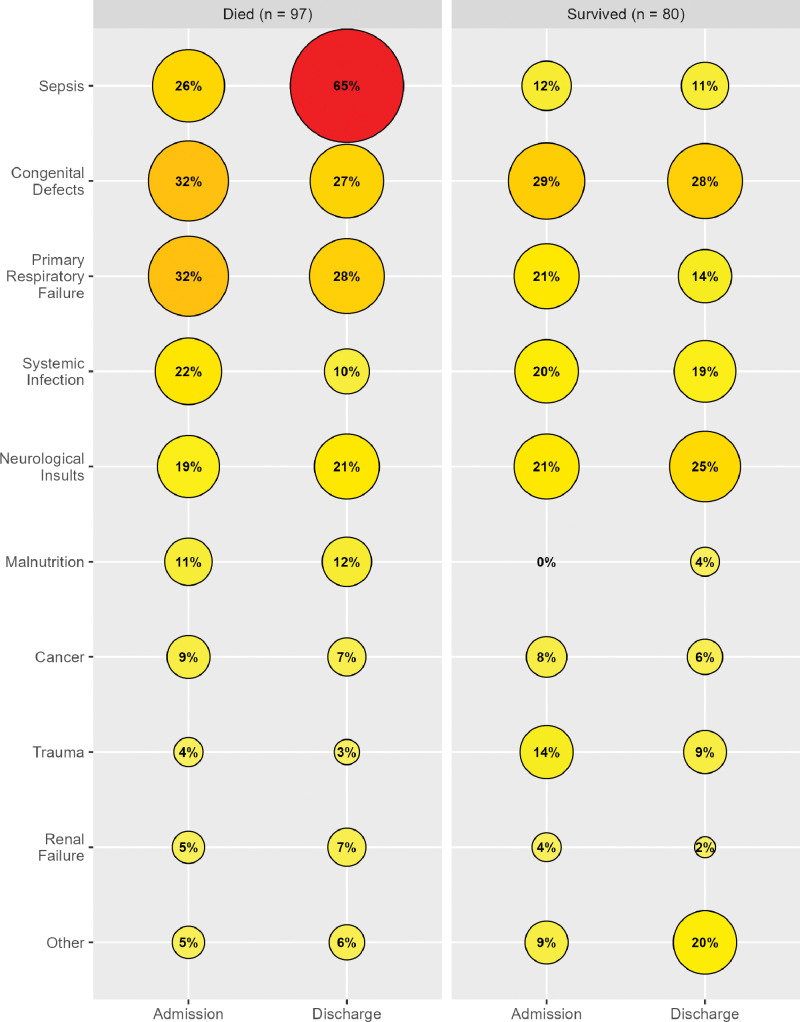
Diagnoses by patient outcome and time.

Diagnoses not only varied between admission and discharge, but also based on mortality. Even at admission, sepsis was more common in patients who died than in those who did not. This became more extreme at the time of discharge or death, with 65% of patient visits that resulted in death having sepsis listed as one of the diagnoses. Similarly, malnutrition was often listed in addition to another diagnosis and was more likely to result in death. To a lesser extent, congenital defects and primary respiratory failure were more common in patients who died. Trauma had the opposite relationship, with more patients surviving if they were admitted for trauma‑related injuries (Figure 1).

### Medication

Examining commonly prescribed medications provides insight into resource availability. Most patients were prescribed multiple medications and medication types during their stay in the PICU. Antibiotics were the most prescribed type of medication, with 91% (*n* = 161) of patient visits utilizing an antibiotic. Of the antibiotics prescribed in the PICU, cefotaxime, meropenem, cloxacillin, and vancomycin were the most common (*n* = 76, *n* = 71, *n* = 64, *n* = 60, respectively). Sedatives were also commonly prescribed with 62% of patient visits requiring a sedative. Similarly, antipyretics and nutritional electrolyte supplements (NES) were prescribed 54% and 46% of the time.

### Factors affecting mortality

Logistic regression techniques were used to investigate the relationship between information available at admission and mortality. Univariable logistic regression indicated admittance delay (log(OR) =1.49 95% CI: [0.46, 2.76] *p* = 0.009), surgery (log(OR) = −0.88 95% CI: [−1.52, −0.26] *p* = 0.006), age (log(OR) = −0.01 95% CI: [−0.0122, −0.0003] *p* = 0.04), and weight (log(OR) = −0.03 95% CI: [‑0.06,3 −0.004] *p* = 0.03) had a significant relationship with mortality. Therefore, they were considered for inclusion in the multivariable model. However, age and weight were significantly correlated (*p* < 2.2 × 10^−16^), so only age was used in the multivariable model since it was more interpretable.

**Figure 2:**
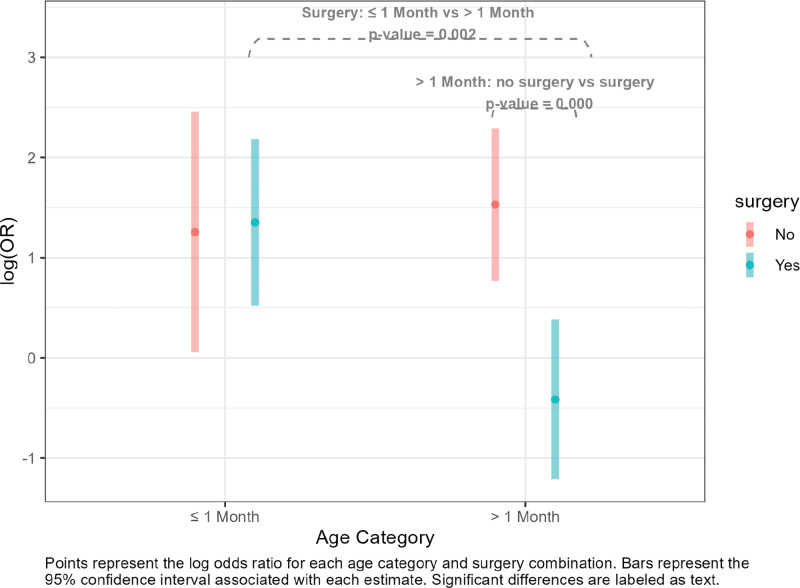
Interaction between surgery and age.

All variables maintained their significance in the multivariable model, including the interaction between age and surgery (Table 4). Holding all other predictor variables constant, patients with a noted delay in admittance to the PICU had increased odds of mortality. Holding delay in admittance constant, newborns (one month or younger) who had surgery had higher odds of mortality than non‑newborns with surgery. Also, non‑newborns who did not have surgery had higher odds of mortality than non‑newborns who did have surgery (Figure 2, Table S1).

**Table 4:** Multivariable logistic regression results.

CHARACTERISTICS	LOG(OR)	95% CI	*P*‑VALUE
Admittance Delay			
No	—	—	
Yes	1.7	0.57, 3.1	**0.006**
Age Category			
≤1 Month	—	—	
>1 Month	0.28	−0.93, 1.4	0.6
Surgery			
No	—	—	
Yes	0.10	−1.2, 1.3	0.9
Age Category * Surgery			
>1 Month * Yes	−2.0	−3.6, −0.52	**0.008**

Abbreviations: CI = Confidence Interval, OR = Odds Ratio

Of the admitting diagnoses, sepsis was associated with higher odds of mortality (log(OR) = 0.90 95% CI: [0.10, 1.78] *p* = 0.03) and trauma was associated with lower odds of mortality (log(OR) = −1.42 95% CI: [−2.74, −0.30] *p* = 0.02).

## Discussion

Our findings show expected consistency with other studies conducted in LMIC with a mortality of 55% of pediatric patients admitted to the PICU [11, 10, 27]. Severity of illness and comorbidities of children prior to entry into the PICU foretell mortality. Additionally, the time between symptom onset and hospitalization can affect outcomes. Because congenital malformations are often evident at birth, the interval between presentation of symptoms and hospitalization was often shorter in that cohort. Furthermore, some infants with congenital birth defects need immediate surgery to sustain life. While there was not a significant relationship between the time between symptom onset to hospitalization and mortality, this information was missing 14% of the time, which could have affected the results. Conversely, longer transfer times may indicate symptoms worsened over time or that something prevented the patient from being transferred, but additional details would be needed to make that distinction. Once admitted to the PICU, shorter stay lengths were in part due to deaths occurring soon after admission.

All children entering the PICU required intubation and ventilatory support. If closer surveillance of early decompensating respiratory status was possible, and access to noninvasive respiratory support were more accessible, the need for PICU care might have been aborted.

Infants under one month were more vulnerable to mortality than older children, perhaps because they arrived with critical congenital defects such as cyanotic heart defects or omphalocele requiring immediate supportive ventilation and aggressive nutritional support. While specialized pediatric surgeons are available, they are more limited in the country, and this could account for a delay in transport to the theater until a specialized team of surgeons and pediatric equipment could be gathered. Conversely, older children who required surgery after accidents had a lower mortality rate, suggesting available trauma surgeons and a more stable overall health state prior to the accident.

Over 50% of the admitted patients were prescribed nutritional electrolytes and supplements, implying vitamin deficiency or malnutrition as comorbidities, and many were diagnosed with malaria, suggesting either acute illness or chronic malaria.

We identified sepsis as one of the most common admitting diagnoses and identified cause of death. However, sepsis was often included with additional diagnoses, making it difficult to interpret its individual effect. While there are well‑established guidelines for diagnosing and treating sepsis in the adult population [17], global agreement in pediatrics has yet to be established [18, 20, 21]. In addition, defining sepsis has traditionally required advanced hemodynamic monitoring often not available in LICs. A consensus was established by a panel of global experts in a recent paper on the management of adult sepsis in resource‑limited settings [23], but these guidelines are not applicable to infants and children. Still, there is agreement among pediatric intensivists that the administration of vasoactive medications, timely administration of antibiotics, and rapid infusion of isotonic fluids are critical to reducing mortality [24, 25]. Thus, any delay in treatment is a critical metric.

The most current WHO list of essential medicines for children has limited vasoactive medications for inotrope support needed to survive sepsis [26]. As an example, milrinone, a powerful inotrope and lusitrope to treat low cardiac output from septic shock, is used extensively in HICs. However, it is not included in the latest 2021 WHO essential drugs impacting the reversal of sepsis.

Of the antibiotics prescribed in the PICU, the researchers noted cefotaxime, meropenem, cloxacillin, and vancomycin were the most common. These are broad‑spectrum antibiotics often used for drug‑resistant organisms. Microbiological analysis, such as culture and sensitivity of blood samples, was not always available. When it was, antibiotics were prescribed accordingly. However, there were limited essential medications, including narrow‑spectrum antibiotics, risking the development of drug‑resistant organisms both in the PICU and the larger community. Amoxicillin, gentamicin, and ampicillin are considered first‑line antibiotics in treating severe sepsis and severe community‑acquired pneumonia in neonates and children [22]. However, a study conducted in 2020 on the availability of antibiotics and other medications throughout Kigali city and five other districts in Rwanda found that the availability of antibiotics in the public and faith‑based sector was poor, particularly for amoxicillin [28].

## Limitations and Bias

Most data were gathered directly from handwritten charts without the benefit of computerized medical records. Consequently, challenges during the data collection process included linguistic barriers, legibility of handwriting, missing files, inconsistency in patient identification numbers, and incomplete or missing information. While English was used predominantly throughout the files, on occasion, French and the native language Kinyarwanda were interspersed. These restrictions may have altered the data slightly and could have changed some of the final tallies. Longer text fields, like the diagnosis information, could have been impacted more strongly. Additionally, the variable of delay in admission required written documentation of a delay in the record. It is possible delays occurred in other patient encounters but were not documented as such. Furthermore, the notation of delay did not clearly define the reasons or type of delay, making interpretation inexact. The initial selection of 30 charts per year was based on subject matter expertise and practical limitations while collecting the data. As a result, we were able to capture 177 of 996 (17.78%) of all patients in our sampling period. Due to the use of random sampling from an annual cohort, results may be slightly skewed by not equally accounting for seasonal illness throughout the year. Even though a sample size calculation was not performed, the one in ten rule was used as a guideline for limiting the number of predictors in the multivariable regression model [29]. Interaction terms are more difficult to detect; however, it was maintained in the model since it had a clinical interpretation.

## Future Direction

This study identified variables impacting mortality in one PICU in a low‑income country. Extracting more details on each of the variables, particularly delays, time between symptom onset and hospitalization, and combinations of diagnoses, would help to develop plans for best practice and improving survival. Attention to the vulnerability of the noticeably young child could provide key answers to their higher mortality. Also, examining factors related to additional outcomes, such as length of stay or readmittance rate, could provide more insights. Strategies impacting global health policies for ensuring essential supplies, along with collaborative partnerships for specialized education, should be examined to engage a wider community of global health providers.

## Conclusion

To reduce mortality in critically ill children in low‑income settings, developing strategies to reduce delays in admitting children to the PICU, increasing bed capacity with associated specialized education for medical and nursing staff, and following emerging guidelines for sepsis in children are necessary. These strategies will take collective efforts by the global health community to influence policy changes, particularly in less resourced settings where critical disparities exist. Equalizing care for critically ill children worldwide ought to be a global health priority.

## Additional Files

The additional files for this article can be found as follows:

Supplementary Table 1. Post hoc analysis of surgery and age interaction.
