# Supplementary material for: Resource Allocation in the Pediatric Intensive Care Unit in Rwanda
Source: Ann Glob Health. 2025 Aug 26;91(1):48. doi: 10.5334/aogh.4714 (PMC12396189; doi:10.5334/aogh.4714)
Supplement: Supplementary Table 1. — Post hoc analysis of surgery and age interaction. [file agh-91-1-4714-s1.pdf]

Table S1: Post-hoc analysis of surgery and age interaction

Group 1		Group 2				
Age	Surgery	Age	Surgery	Estimate	95% CI	p-value
≤ 1 Month	No	> 1 Month	No	-0.276	-1.80, 1.25	0.967
≤ 1 Month	No	≤ 1 Month	Yes	-0.096	-1.70, 1.51	0.999
≤ 1 Month	No	> 1 Month	Yes	1.671	0.01, 3.33	**0.047**
> 1 Month	No	≤ 1 Month	Yes	0.180	-0.93, 1.29	0.976
> 1 Month	No	> 1 Month	Yes	1.947	0.78, 3.11	**0.000**
≤ 1 Month	Yes	> 1 Month	Yes	1.767	0.50, 3.03	**0.002**

Results are averaged over the levels of admittance delay and given on the log odds ratio scale.

Conf-level adjustment: tukey method for comparing a family of 4 estimates.
