# Supplementary material for: Resource Allocation in the Pediatric Intensive Care Unit in Rwanda
Source: Ann Glob Health. 2025 Aug 26;91(1):48. doi: 10.5334/aogh.4714 (PMC12396189; doi:10.5334/aogh.4714)
Supplement: Supplementary file 2 — Supplementary File 1. [file agh-91-1-4714-s2.pdf]

Supplemental

Words for diagnoses:

To group the diagnoses into categories, certain words associated with each category were looked for in the diagnoses fields. If that word was found, the patient visit was associated with that diagnosis category. The list of diagnoses and their associated words are listed below.

Trauma: burn, trauma, polytrauma, fracture, crush, wound, laceration, organophosphate ingestion, splenic liver injury, tbsa

Sepsis: sepsis, septic, shock, hypovolemic shock, nns, disseminated staphylococal infections, neonatal infection, disseminated infection, multi-organ dysfunction

Cancer: cancer, tumor, leukemia, medulloblastoma, astrocytoma, neck mass, neoplastic syndrome, neuroblastoma, medulloblastoma, lymphoma

Congenital defect: congenital, gastroschisis, atresia, fistula, abdominal wall defect, anorectal malformation, anorectal, cdh, chf-vsd, cloaca malformation, craniopharyngioma obstructive hydrocephalus, congenital heart defect, diaphragmatic hernia, esophageal stenosis, hirschbrungs disease, hirschsprung's disease, asd, polydactyl, gastoschisis, pda, space occupying legions, interrupted aortic, tef, tef/, umbilical hernia repair, vsd, duodenal stenosis, duodenal web, umbilical cord hernia repair

Renal failure: aki, acute kidney injury, chronic kidney disease, hemolytic uremic syndrome, nephrotic

Primary respiratory failure: respiratory, pneumonia, pneuomonia, pneuomnia, parapneumonic, bronchitis, bronchiolitis, bronchopneumonia, foreign body in lungs, rf , tracheobronchal foreign body, meconium aspiration, pleural effusion, pneumothorax, supraglottic stenosis

Neurological insult: tbi, cerebral malaria, meningoencephalitis, meningitis, cerebral edema, coma, brain, head, convulsion, diffuse oxonal injury, stroke, intracranial, hydrocephalus, intracerebral, pneuomocephalus, craniotomy, oxinal, subdural empyema, epilepticus, encephalopathy, seizure, decreased level of consciousness

Systematic infection: malaria, fever, tetanus, gastroenteritis, abscess, empyema, hiv, hsv, pyomyositis, multi-organ dysfunction syndrome, mods, non nutritive sucking, osteomyelitis, peritonitis, endocarditis, tb , arteritis, toxic epidermal necrolysis, typhoid, tuberculosis

Malnutrition: malnutrition, rickets, failure to thrive, inability to breastfeed

Words for medications:

A similar methodology was used to group medications into the following categories.

AI: meropenem, cefotaxime, vancomycin, cloxacillin, ceftriaxone, ampicillin, flagyl, ciprofloxacin, metronidazole, gentamycin, amikacin, cefotaxine, cloxacilline, azithromycin, bactrim, erythromycin, ciprofloxacine, meropenum, piperacillin, cefotriaxone, ceftazidime, ceftrioxone, clamoxyl, polymyxin b, amoxcillin, amoxicillin, ampicilline, ampicilln, augmentin, bronquidiazina, carbapenem, cefazolin, cefotoxime, cefoxitin, cefriaxone, ceftaxime, ceftraixone, ceftriazone, ceprofloxacin, cetotaxime, chlorpheniramine, chlorphreniramin, ciproflaxine, cloaxacillin, cloxacilin, doxacillin, furadantin, glutamycin, imipenem, meropeneum, meroperum, merpoenem, polymyxin, tetracyclin ointment, tetracycline, vancomycine, vancoymycin

AI F: flucanazole, nystatin, clotrimazole, flucanzole, fluconzole, ketoconazole

AI HIV: nevirapine

AI M: artesunate, coartem, antesamate, artesunate amivas, tab coartem

AI P: vermox, albendazole, mebendazole

AI V: fluconazole, gentamicin, acyclovir, aciclovir, acyclovir syrup

AP: paracetamol, ibuprofen, brufen, paracetemol, parecetamol, pct, betafed syrup, brufen syrup, ibuprofen syrup, paracetamol syrup, paracetemamol, parectamol, pct support, profen

AS: phenytoin, diazepam, phenobarbital, depakene, carbamazepine, depakin, depakote, diazepan, phenoboubitous, phenyotin, seizure control with phenobarbital and phenytoin, sodium valproate

Antifibrinolytic tranexamic acid

BLOOD: rbc transfusion

BP: amlodipine, nifedipine, propranolol, hydralazine, atenolol, sildenafil, captopril, hydralyzine, nifedipine tab

D: lasix, furosemide, acetazolamide, mannitol, acetazolamida, diamox, hydrochlorothiazide, lasix:, acetazolamide for fluid overload, aldactone, aldactone tab

Human Blood Product: human albumin, albumin, erythropoietin

Human Product: insulin, insulin rapid, insulin lente, insulin, rapid, depolin, insulin "regular", rapid insulin

NES: calcium gluconate, omeprazole, kcl, vitamin k, cimetidine, potassium chloride, lactulose, calcium glucomate, calcium glutinate, esomeprazole, zinc oxide, calcitriol, gaviscon, zedcal, zinc oxide cream, ameitidine, ascoril syrup, ca2, calcium, calcium glucamate, calcium glyconate, calcium tab, centamin, depanitab, esoculprozol, fercefol, fercefol syrup, fercetol, glycerin, glycerine, glycerine suppo, kcl:, lactulose syrup, mg so, mgso, multivitamin, omprezaole, procalcitonin, sodium chloride, vitamin b, vitamin d, vitamin k:, zedcal syrup, zinc, zinc sulfate, zinc sulphate, znso

ORAL FLUID: ors

Pulmonary: salbutamol, aminophylline, salbutamol nebulizer, amphinophylline, nebulization, nebulization with saline, nebullization with adrenaline, salbutaml, salbutamol nebulization, salbutamol spray, sekrol syrup, theophylline, ventalin, xylometazoline

R: bicarbonate

RI: adrenaline, dopamine, atropine, adrenaline nebulizer, dobutamine, dopamine infusion, epinephrine, adrenaline dose not recorded, adrenaline neb, adrenaline nebulization, noradrenaline

S: fentanyl, morphine, midazolam, ketamine, midozolam, vecuronium, medazolam, depolam, fentinol, ketomine, midazlam, midozolam morphine, moprhine, morphin, morphine syrup, propofol, sedated with midazolam and fentanyl, vecuromium

Steroid: dexamethasone, hydrocortisone, prednisolone, prednisone, dexamethansone, dexmaethasone, dexomethasone, hydrocortisol, methylprednisolone

Vaccine tetanus: immunoglobulin, tetanus toxoid

Chemotx: adriamycin, chemotherapy, cyclophosphamide, vincristine

Lovenox: enoxaparin, levenox, lonox
